# Successful adjuvant-free vaccination of BALB/c mice with mutated amyloid β peptides

**DOI:** 10.1186/1471-2202-9-25

**Published:** 2008-02-18

**Authors:** Chuanhai Cao, Xiaoyang Lin, Monika M Wahi, Eugene A Jackson, Huntington Potter

**Affiliations:** 1 Johnnie B. Byrd Alzheimer's Center and Research Institute, 4001 E. Fletcher Ave., Third Floor, Tampa, FL 33613, USA; 2 University of South Florida College of Medicine, Dept. of Molecular Medicine, 12901 Bruce B. Downs Blvd. MDC 10, Tampa, FL 33612, USA; 3 University of South Florida College of Public Health, Department of Epidemiology and Biostatistics, 13201 Bruce B. Downs Blvd. MDC 56, Tampa, FL 33612, USA

## Abstract

**Background:**

A recent human clinical trial of an Alzheimer's disease (AD) vaccine using amyloid beta (Aβ) 1–42 plus QS-21 adjuvant produced some positive results, but was halted due to meningoencephalitis in some participants. The development of a vaccine with mutant Aβ peptides that avoids the use of an adjuvant may result in an effective and safer human vaccine.

**Results:**

All peptides tested showed high antibody responses, were long-lasting, and demonstrated good memory response. Epitope mapping indicated that peptide mutation did not lead to epitope switching. Mutant peptides induced different inflammation responses as evidenced by cytokine profiles. Ig isotyping indicated that adjuvant-free vaccination with peptides drove an adequate Th2 response. All anti-sera from vaccinated mice cross-reacted with human Aβ in APP/PS1 transgenic mouse brain tissue.

**Conclusion:**

Our study demonstrated that an adjuvant-free vaccine with different Aβ peptides can be an effective and safe vaccination approach against AD. This study represents the first report of adjuvant-free vaccines utilizing Aβ peptides carrying diverse mutations in the T-cell epitope. These largely positive results provide encouragement for the future of the development of human vaccinations for AD.

## Background

Currently, more than five million people in the United States suffer from Alzheimer's disease (AD), and given our aging population, this prevalence is expected to continue to rise [1]. Neuropathologic hallmarks in AD include the formation of toxic amyloid β (Aβ), its aggregation into globular oligomers (plaques) in the brain, and the subsequent formation of a neurotoxic protein leading to cognitive and behavioral deficits and neurodegeneration [2]. One reasonable approach to this future public health crisis is to decrease the incidence and possibly prevalence of AD through the development and administration of a human vaccination that prevents, slows, or removes this AD pathology in human brains.

The first attempt at developing such a vaccine was documented in 1999, when Schenk et al. reported that Aβ1–42, when used as an active vaccine, can effectively remove Aβ plaques in AD transgenic mouse brains [3]. Another milestone vaccine study published in 2000 showed that the use of Aβ1–42 plus adjuvant as an active vaccine in the AD transgenic mouse model not only induced an effective remission of Aβ plaques in the brain [4], but also led to cognitive and behavioral improvements [5]. In other mouse model studies, passive immunotherapy with anti-Aβ antibodies produced similar results [6,7]. Further, Yamamoto et al. demonstrated that antibodies to Aβ1–42 may effectively inhibit the deposition of Aβ in the brain [8]. Weiner et al. also reported plaque-lowering in PDAPP transgenic mice after an intranasal inoculation of Aβ without adjuvant [9].

Mutated peptides have been shown to have different phenotypes. Mutations in the APP gene and the resulting mutant Aβ peptides are highly associated with autosomal dominant AD. The Dutch and Flemish mutations are known to cause patterns of aggregation that strongly differ from those with wild type (Wt) Aβ peptide [10,11]. Further, the Dutch and Flemish mutations have different phenotypes [12]. While both the Dutch and Flemish mutations cause hemorrhage and amyloidosis in patients, only the Flemish mutation causes AD [13,14].

In addition, it is important to consider the possibility that vaccination with Aβ may induce an unwanted inflammatory response. Popovic et al. report that the presence of antigen-presenting HLA-DR-positive and other immunoregulatory cells together with abnormal levels of inflammatory cytokines and acute phase reactants are consistently detected in tissue of AD neuropathology [15]. It has been theorized that AD-related inflammation could be a form of autoimmunity that potentially marks a more specific and progressive state of the disease [16]. Vaccination with an Aβ peptide, therefore, runs the risk of exacerbating this inflammation. And because vaccine adjuvants themselves can cause varying levels of inflammation [17], the effect of the adjuvant is important to consider in the development of an AD vaccine.

The increased success with vaccinations using Aβ peptides in mouse models of AD encouraged a human clinical trial. The study was a randomized, multi-centered, placebo-controlled, double-blind trial using Wt Aβ peptide AN1792 as a vaccine in combination with the adjuvant QS-21 combined with polysorbate-80 [18,19]. The trial included patients aged 50 to 85 years of age with probable AD as determined by the Mini-Mental State Examination (MMSE) [20]. Unfortunately, meningoencephalitis occurred in 6% of the 298 participants, forcing this Phase II trial to be suspended by the United States Food and Drug Administration. However, in a follow-up study of the vaccinated patients, some benefits were seen, including reduced AD-like pathology on autopsy [21] and improved cognition [22]. While this study suggests that a vaccination with an Aβ peptide may be helpful in humans, more work must be done to develop one that does not produce such serious side effects.

The cause of the adverse events in the AN1792 trial has not been identified, and further analysis is required to determine the mechanism of neuroinflammation and the associated meningoencephalitis. As part of the analysis, it is necessary to consider that the inflammatory response might actually have been triggered, at least in part, by the adjuvant, and not the antigen [17,23]. It has been proposed that the polysorbate-80 that was added to the QS-21 adjuvant to keep it from precipitating out may have contributed to the meningoencephalitis in the AN1792 trial [18]. In fact, other studies have shown that adjuvants themselves induce significant pro-inflammatory cytokine expression *in vivo *including up-regulation of TNF-α, IFN-γ, and IL-4, even without being coupled to an antigen [23]. Immune modulation may also be affected by adjuvant administration, and this must be taken into account when selecting a particular adjuvant. Importantly, this modulation may result in the production of different antibody subclasses [24]. These negative properties associated with adjuvants suggest that an adjuvant-free vaccine for AD would be an attractive prospect.

Historically, the use of an adjuvant in vaccination was necessary to prime the immune system. However, our research group recently reported that mutations in the Aβ1–42 peptide can change the antigenicity of the peptide [10]. This was further supported by a study by Arendash et al. which demonstrated that adapted transfusion of Aβ-peptide-specific T-cells into AD transgenic mice can reverse cognitive impairment, suggesting that T-cell activation may be necessary for Aβ clearance [25]. The Aβ peptide itself contains two strong B-cell epitopes, as well as a strong T-cell epitope [10]. Previous studies suggested that adjuvant-free vaccination might help identifying pathways of autoimmunity [26]. These findings suggest that the Aβ peptide itself may be able to serve as its own adjuvant.

One of the participants in the AN1792 trial was found to have multiple cortical hemorrhages upon autopsy [27]. Indeed, mouse model studies have suggested that hemorrhage may be an adverse event of Aβ vaccination that must be considered in the development of human Aβ vaccines [28-30]. The mechanism behind hemorrhage post-Aβ clearance has been proposed to be due to weakening of the vessel walls [28]. We believe that a vaccine that causes Aβ clearance in the brains of older human patients can still be developed; however, the clearance should happen at a slow, steady rate to allow the vessel walls to acclimate to the removal of the plaque. This suggests that an appropriate vaccine should not induce a strong, quick immune response. Rather, a good vaccine for the purpose of Aβ plaque removal would demonstrate a slow increase in antibody that held at stable levels for a long duration in which memory response could be demonstrated. This is in contrast to the goal of prophylactic vaccines, which is to induce a quick and strong immune response in the vaccine.

The current scientific goal is to build upon our understanding of the results of the AN1792 trial to find a safer and more effective vaccine candidate. The aim of this study is to test adjuvant-free vaccines using Aβ peptides, both Wt and those with mutations in the T-cell epitope, and evaluate their efficacy and safety. Here, we report the successful vaccination of BALB/c mice with several adjuvant-free mutated Aβ peptides.

## Results

### Antibody response

**Figure 1:**
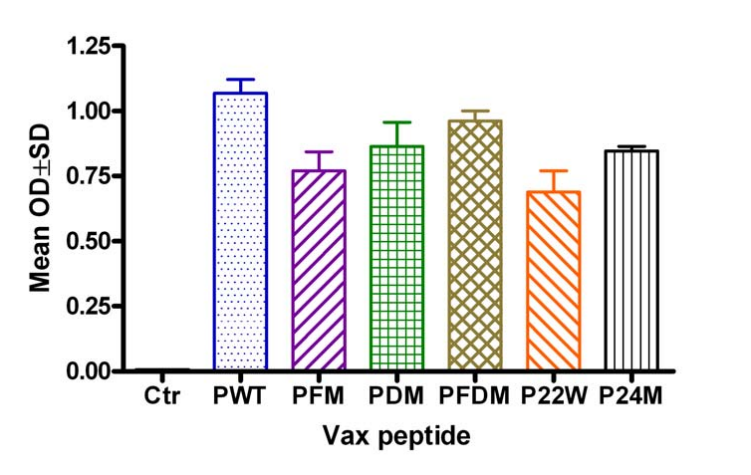
**Antibody detection after 3 inoculations with different Aβ peptides**. Note: ELISA result for antibody detection at 1:1024 dilutions using Aβ1–42 wild type as capture antigen at 10 μg/ml (50 μl/well) (n = 4 in each group). Ctr = Control, PWT = Wild type Aβ1–42, PFM = Aβ1–42 with Flemish mutation, PDM = Aβ1–42 with Dutch mutation, PFDM = Aβ1–42 with Flemish and Dutch mutation, P22W = Aβ1–42 with novel mutation at 22, P24M = Aβ1–42 with novel mutation at 24 amino acid.

**Figure 2:**
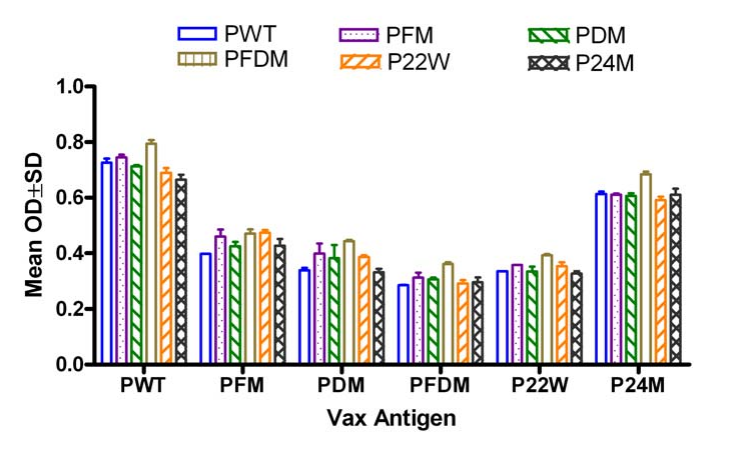
**Cross-reaction analysis of binding to various Aβ peptides**. Note: ELISA result for antibody detection at 1:1024 dilutions using different Aβ1–42 peptides as capture antigens at 10 μg/ml (50 μl/well). There are good peptide cross recognitions (n = 4 in each group). PWT = Wild type Aβ1–42, PFM = Aβ1–42 with Flemish mutation, PDM = Aβ1–42 with Dutch mutation, PFDM = Aβ1–42 with Flemish and Dutch mutation, P22W = Aβ1–42 with novel mutation at 22, P24M = Aβ1–42 with novel mutation at 24 amino acid.

**Figure 3:**
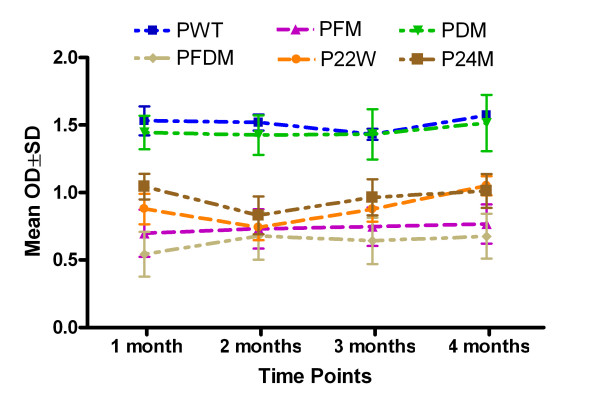
**Antibody duration after 3 inoculations at 1, 2, 3, and 4 months**. Note: ELISA results for antibody detection using Aβ1–16 as capture antigen at 20 μg/ml (50 μl/well), plasma were diluted at 1:2048 dilutions. There is no statistically significant reduction four months after vaccination. PWT = Wild type Aβ1–42, PFM = Aβ1–42 with Flemish mutation, PDM = Aβ1–42 with Dutch mutation, PFDM = Aβ1–42 with Flemish and Dutch mutation, P22W = Aβ1–42 with novel mutation at 22, P24M = Aβ1–42 with novel mutation at 24 amino acid.

**Figure 4:**
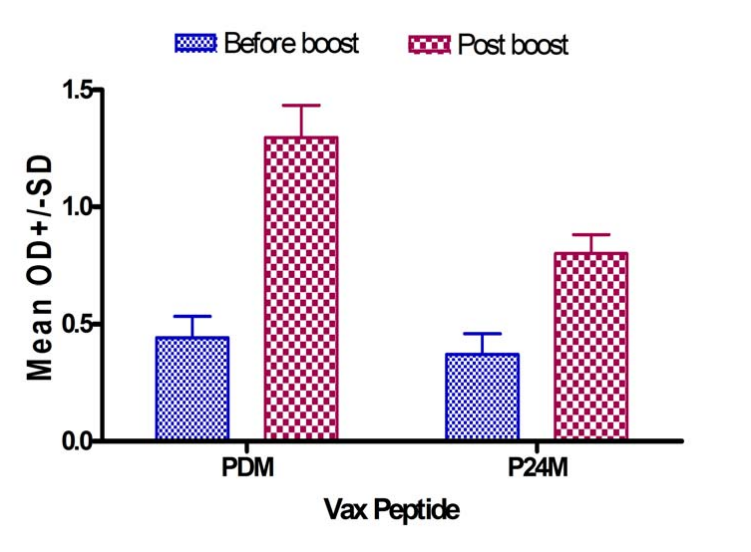
**Immune memory response following booster injection**. Note: ELISA results for antibody detection using Aβ1–16 as capture antigen at 10 μg/ml (50 μl/well) and plasma diluted at 1:3200 dilutions. Blue bar shows antibody detection 6 months after third inoculation and just prior to boost, and red bar shows 10 days after boost. PDM = Aβ1–42 with Dutch mutation, P24M = Aβ1–42 with novel mutation at 24 amino acid.

Mutated and Wt Aβ1–42 peptide vaccinations induced clear antibody responses after 2 inoculations (data not shown) and high antibody level after 3 inoculations, while no antibody could be detected in the control group (Figure 1). There was a high antibody response 10 days after the third vaccination for all peptide vaccine groups at 1:1024 dilutions, and antibody titers rose to greater than 1:4096 in all peptide vaccine groups (data not shown). There were no differences in peptide recognition among various anti-sera and peptides, with the exception of Wt (PWT) and P24M (novel mutation), which were higher regardless of coating antigen (Figure 2). Antibody was long-lasting, showing duration of up to 4 months (Figure 3). Memory response was also apparent when comparing antibody levels before and after boosting (Figure 4).

In summary, all peptides used in this study induced good antibody responses. However, PFM (Flemish mutation), PFDM (Dutch and Flemish mutation) and P22W (novel mutation) had better antibody duration responses.

### Epitope Mapping

**Figure 5:**
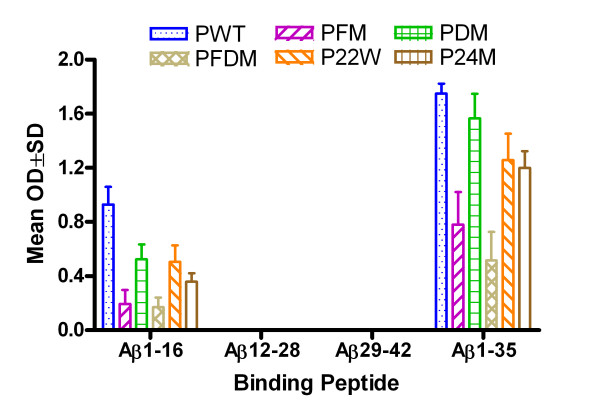
**Epitope mapping of anti-sera using various Aβ fragments**. Note: ELISA results for epitope mapping using various Aβ peptide fragments as capture antigens at 20 μg/ml (50 μl/well) and plasma were diluted at 1:1024 dilutions. PWT = Wild type Aβ1–42, PFM = Aβ1–42 with Flemish mutation, PDM = Aβ1–42 with Dutch mutation, PFDM = Aβ1–42 with Flemish and Dutch mutation, P22W = Aβ1–42 with novel mutation at 22, P24M = Aβ1–42 with novel mutation at 24 amino acid.

**Figure 6:**
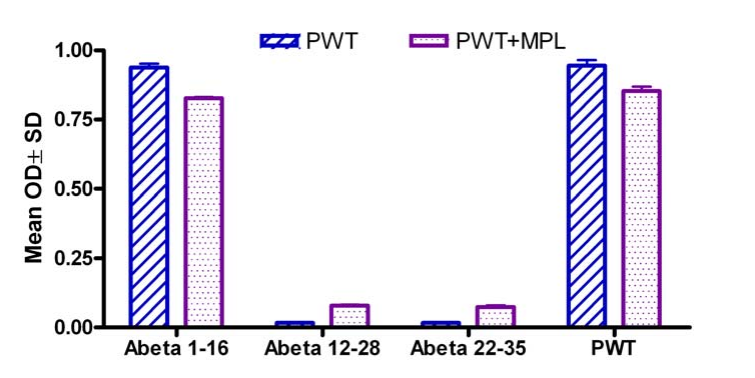
**Comparison of epitope mapping of Aβ peptide with and without adjuvant using various Aβ fragments**. Note: ELISA results for epitope mapping using various Aβ peptide fragments as capture antigens at 20 μg/ml (50 μl/well) and plasma diluted at 1:1024 dilutions. Blue bar represents non-adjuvant vaccine (current study), while purple bar represents results from a previous adjuvant study [10]. PWT = Wild type Aβ1–42, PWT+MPL = Wild type Aβ1–42 mixed with monophosphoryl lipid A (MPL) as an adjuvant.

All antibodies induced by the different peptides injected demonstrated binding to Wt Aβ1–16 and 1–35 (Figure 5). Figure 6 shows non-adjuvant epitope mapping in blue (data from the current study) compared to adjuvant vaccine epitope mapping in purple from a previously published study [10]. In this previous study, MPL+TDM (Sigma Aldrich, MO) was used as an adjuvant because Freud was prohibited for use in vaccine studies in mice, and MPL+TDM is water soluble (thus preventing aging of the peptide), avoids emulsification of the peptide, and preserves the antigen [31]. MPL has been demonstrated to drive a Th1 response in mice [32,33] and performed similarly in our earlier study as in other vaccine studies using this adjuvant [34]. It is apparent there is no difference in epitope between the adjuvant and non-adjuvant. Toellner et al has reported a study that cautions that mutant peptides may be subject to epitope switching [35]. Our results indicate that there was no epitope switching among mutations, and additionally, there were no differences in epitope when compared to the adjuvant vaccine.

### Cytokine Expression, T-cell Response and IgG Isotyping

**Figure 7:**
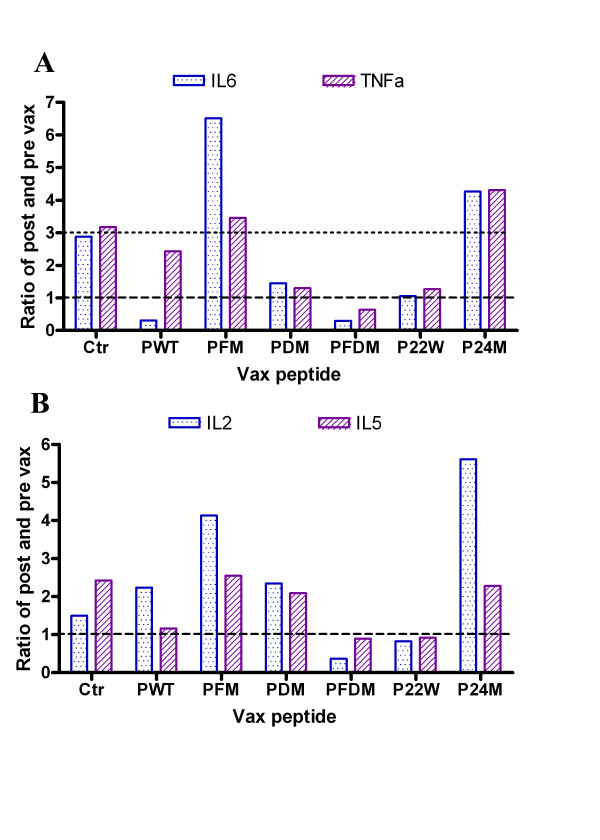
**Cytokine response to vaccination with various Aβ peptides: IL-6, TNF-α, IL-2, and IL-5**. Note: Ratio of post- to pre-vaccination cytokine levels are shown. Ctr = Control, PWT = Wild type Aβ1–42, PFM = Aβ1–42 with Flemish mutation, PDM = Aβ1–42 with Dutch mutation, PFDM = Aβ1–42 with Flemish and Dutch mutation, P22W = Aβ1–42 with novel mutation at 22, P24M = Aβ1–42 with novel mutation at 24 amino acid. Top figure (7a) shows results for IL-6 and TNF-α and bottom figure (7b) shows results for IL-2 and IL-5.

**Figure 8:**
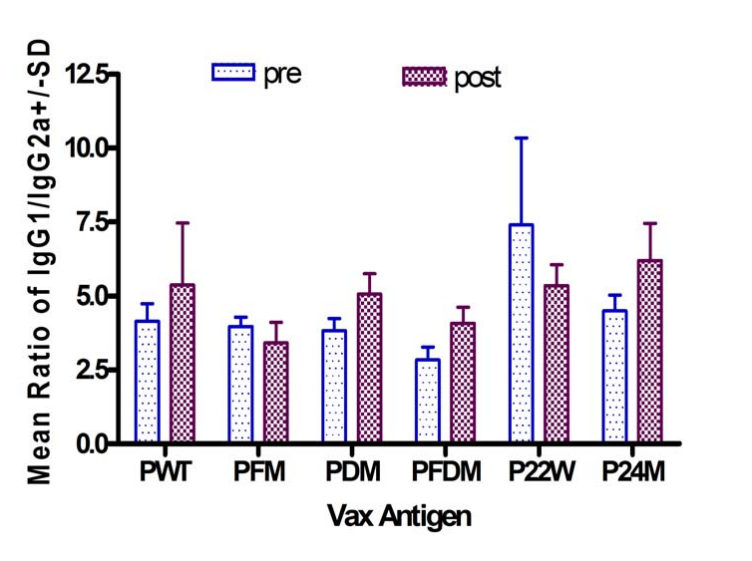
**IgG1/IgG2a ratio pre- and post-vaccination with various Aβ peptides.** Note: As measured by Beadlyte^® ^Mouse Immunoglobulin Isotyping Kit; Wild type Aβ1-42 = Wild type Aβ1–42, Aβ1-42 with Flemish mutation = Aβ1–42 with Flemish mutation, Aβ1-42 with Dutch mutation = Aβ1–42 with Dutch mutation, Aβ1-42 with Flemish and Dutch mutation = Aβ1–42 with Flemish and Dutch mutation, Aβ1-42 with novel mutation at 22 = Aβ1–42 with novel mutation at 22, Aβ1-42 with novel mutation at 24 amino acid = Aβ1–42 with novel mutation at 24 amino acid.

Several peptides induced low levels of IL-6, TNF-α, IL-2, and IL-5 (Figures 7a and 7b). All peptides except P22W produced an IgG1/IgG2a ratio that was either higher or no different post-vaccination than pre immunization. This indicates a high Th2 response (see Figure 8). Also per Figure 8, PDM, PFDM and P24M demonstrated a Th2 type antibody response.

**Figure 9:**
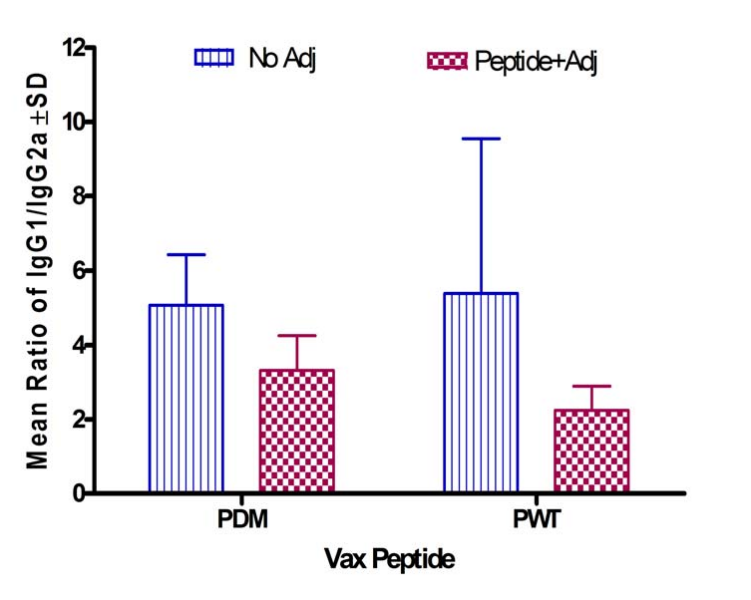
**Comparison of IgG1/IgG2a ratio post-vaccination of wild type and Dutch mutation Aβ peptides with and without adjuvant**. Note: As measured by Beadlyte^® ^Mouse Immunoglobulin Isotyping Kit; PWT = Wild type Aβ1–42, PDM = Aβ1–42 with Dutch mutation. Striped bars report results from current study; checkered bars report results from a previous study [10].

We also compared Wt and Dutch mutation IgG1/IgG2a ratio with data from a previous study [10] to compare Th1 or Th2 response to peptides administered with adjuvant. As shown in Figure 9, non-adjuvant vaccine induced a higher Th2 response compared to adjuvant vaccine in both Wt and Dutch mutation peptides.

In conclusion, some of the non-adjuvant peptide vaccines were directed towards a Th2 response (Figure 9), and also showed a peptide-specific response (Figure 8). PDM, PFDM and P22W showed lower pro-inflammation response (Figures 7a and 7b).

### Anti-sera cross-reactivity

**Figure 10:**
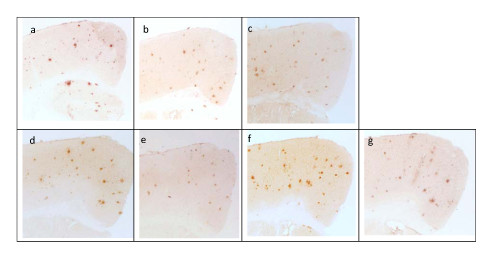
**Cross reaction of antisera from BALB/c mice vaccinated with different adjuvant-free peptide vaccines to cortex brain section of a 14-month-old APP/PS1 transgenic mouse**. Each panel is described below: a. Immunostaining with 6E10 antibody (Signet; Philadelphia, PA). b. Immunostaining with antisera from BALB/c mice vaccinated with PFM peptide. c. Immunostaining with antisera from BALB/c mice vaccinated with P22W peptide. d. Immunostaining with antisera from BALB/c mice vaccinated with P24M peptide. e. Immunostaining with antisera from BALB/c mice vaccinated with PFDM peptide. f. Immunostaining with antisera from BALB/c mice vaccinated with PDM peptide. g. Immunostaining with antisera from BALB/c mice vaccinated with PWT peptide

Figure 10 shows the results of immunostaining of cortex tissue from a human APP/PS1 transgenic mouse. Anti-sera from BALB/c mice in each vaccination group was added to the tissue; each panel represents a different vaccination group. Per the figure, binding to plaques was demonstrated for each vaccination group.

## Discussion

We found several adjuvant-free vaccine candidates that produced results suggesting both efficacy and safety. Several candidates demonstrated a good antibody response that lasted at least 4 months (Figure 3), and showed good memory response after boost (Figure 4). These findings are consistent with the AN1792 follow-up findings by Hock et al. in which sustained increases in serum antibodies against Aβ were demonstrated [22]. Epitope mapping was shown to be no different between our non-adjuvant and other adjuvant-containing vaccines, and Th2 response was observed among some of the non-adjuvant peptides tested. These results are encouraging, suggesting we have identified peptides that may be effective for human vaccination.

In terms of safety, in our study of non-adjuvant peptide vaccines, inflammation levels varied minimally among the groups of mice vaccinated with mutated Aβ peptides, and those levels differed from controls as well as levels in mice vaccinated with Wt peptide. The cytokine profiles for mutated peptide vaccinated mice indicated a peptide-specific response with elevated Th1 (TNF-α, IL-2) and Th2 (IL-5 and IL-6) associated cytokines. Cytokine profiles in our study showed peptide-specific differences with respect to markers of pro-inflammation, such as TNF-α and IL-6 (Figure 7a). Some mutated peptides induced an anti-Aβ antibody IgG1 response (PWT and P22W), while others induced an IgG2a response, such as PDM, PFDM and P24M (Figure 8); this can be compared to PWT and PDM with adjuvant vaccine, which induced a significant Th1 response (Figure 9). In addition, pro-inflammatory cytokine levels were not correlated with antibody titers (Figures 1 and 2). Given that the Th2 response to the adjuvant-free vaccines in our study was higher when compared to adjuvant vaccine response, and that there was no evidence of these peptides inducing unwanted inflammation, avoiding the use of an adjuvant may be beneficial. If a poor Th1 response was part of the reason for the meningoencephalitis in the AN1792 trial, using a non-adjuvant vaccine with one of the candidate peptides used in this study would be a reasonable approach.

One of the major concerns regarding vaccination with mutated peptides is epitope switching [35-37]. Our results show that all peptides in the presence or absence of adjuvant map to the same epitope (Figure 6), suggesting that this was not a concern in our study.

Our study has several strengths. An important strength of our study is that we tested several peptides and identified several candidates for further study. T-cell response was indeed variable across the peptides tested, but our results offer the choice of peptides for further study based upon these data. For example, PDM and PFDM demonstrated a low inflammatory response (Figures 7a and 7b), but a high Th2 response (Figure 8). We also demonstrated that, with these vaccinations, an adjuvant is not necessary to achieve the desired responses.

On the other hand, our study also has limitations. Like the AN1792 vaccination, the peptides used in our study contained the entire T-cell epitope. Using peptides that include only part of the T-cell epitope might improve the desired inflammatory response to the vaccine, and should be a subject of future study. In addition, although we measured cytokine expression and conducted antibody isotyping, we did not actually measure T-cell responses. Results from our previous study suggested that the T-cell epitope is related to haplotype [10], so we will conduct T-cell epitope mapping when we conduct this study in a transgenic mouse model. Finally, our study was done only in BALB/c mice, so the results need to be confirmed by conducting a similar study in APP transgenic mice. Since there is a high level endogenous Aβ produced in transgenic mice, immune response may be different compared to BALB/c mice [38]. It is also unclear how the results may translate to other animals and to humans.

## Conclusion

Our study demonstrated that an adjuvant-free vaccine with different Aβ peptides can be an effective and safe vaccination approach against AD. Our adjuvant-free vaccines induced a good antibody response without stimulating an unwanted inflammation reaction. Furthermore, mutations in the T-cell epitope did not affect the B-cell epitope, but generated different levels of inflammation response. Our results provide several mutant Aβ peptides as viable candidates for further study of adjuvant-free vaccines. Further studies in which the entire T-cell epitope is not contained in the Aβ peptide are also warranted, as this may further address inflammatory reactions.

This study represents the first report of adjuvant-free vaccines utilizing Aβ peptides carrying diverse mutations in the T-cell epitope. These largely positive results provide encouragement for the future of the development of human vaccinations for AD.

## Methods

### Animals

Ten-week-old female BALB/c mice were obtained for the study from Jackson Laboratories (Bar Harbor, ME). It is recognized that BALB/c mice "demonstrate Th2-biased immune responses" [39]. Because we were testing novel mutant peptides, and because AD itself is associated with a Th1 response, we felt BALB/c mice were the most appropriate choice for this study.

One 14-month-old APP/PS1 transgenic (tg) mouse was used for immunostaining studies. The mouse was provided by the Florida Alzheimer's Disease Research Center Mouse Behavior and Neuropathology Core.

The mice were housed in Varian standard cages including amber igloos. Prior to initiation of the study, approval was obtained from the Institutional Animal Care and Use Committee (IACUC) at our institution.

### Vaccination

Aβ1–42 peptides were obtained from Synpep (Dublin, CA) based on human Aβ1–42 peptide sequence. Peptides for vaccination were reconstituted to 10 mg/ml in DMSO and further diluted to 2 mg/ml with phosphate buffered saline. Aβ peptide is highly hydrophobic, so we used DMSO (per manufacturer's instructions) to reconstitute it and keep it at a high concentration. We acknowledge that DMSO has been used in previously tested vaccines and may induce an immune response [40-43]. However, we conducted a pilot study in which we injected a human immunodeficiency virus (HIV) peptide into BALB/c mice using the same amount of DMSO as an adjuvant in one group, and HIV peptide using MPL+TDM (Sigma Aldrich; St. Louis, MO) as an adjuvant in another group. The peptide plus DMSO did not induce an antibody response, but peptide plus MPL did (data not shown). Therefore, we concluded that DMSO would not induce an antibody response against the injected peptide in this study. For this reason, although DMSO has been used in other studies as an adjuvant, we do not consider it an adjuvant in this study.

Aβ1–42 peptides were not aged prior to injection. This is because we believe that aggregated Aβ peptide will inhibit epitope presentation compared to unaggregated peptide. In addition, Aβ aggregation in the brain as part of AD is considered a main cause of inflammation seen in these patients [44], so avoiding the injection of aggregated Aβ should reduce the risk of inducing unwanted inflammation.

Twenty-eight (28) mice were divided into 7 groups, each containing 4 mice. Six (6) different mutated Aβ1–42 peptides were used in the study. Each group received one of the 6 peptides, with the final group receiving PBS containing 10% DMSO to serve as a control group.

An initial subcutaneous vaccination was given at 100 μg peptide in 100 μl when mice were 14 weeks old. The next booster vaccination was given 2 weeks later, at 100 μg peptide (100 μl). Finally, 2 weeks later, a third inoculation was carried out with 50 μg peptide in 100 μl.

### Peptide selection

We had decided *a priori *to include Aβ1–42 Wt, Aβ1–42 with Dutch mutation, and Aβ1–42 with Flemish mutation in the study to build upon already published reports [11,12], as well as a control group. Therefore, we were left with enough resources to test 3 additional peptides.

To select these 3 peptides, we took Wt Aβ1–42 and created 100 candidate peptides from introducing mutations at various places. Next, we conducted HLA peptide motif analysis [45] to assist us in choosing peptides. We decided to choose 1 peptide with high affinity, 1 with medium affinity, and 1 with low affinity. "High affinity" was considered as having an HLA affinity score >4,000, "medium" was designated as having a score equal to Wt score (which was between 400 and 1,000), and "low" was considered <100. The sequences for the peptides we chose are given in Table 1.

**Table 1:** Peptides sequences.

Peptide name	Sequence of each peptide
Aβ1–42 Wild type (PWT)	DAEFRHDSGYEVHHQKLVFFAEDVGSNKGAIIGLMVGGVVIA
Aβ1–42 with Flemish mutation (PFM)	DAEFRHDSGYEVHHQKLVFF**G**EDVGSNKGAIIGLMVGGVVIA
Aβ1–42 with Dutch mutation (PDM)	DAEFRHDSGYEVHHQKLVFFA**Q**DVGSNKGAIIGLMVGGVVIA
Aβ1–42 with Flemish and Dutch mutation (PFDM)*	DAEFRHDSGYEVHHQKLVFF**GQ**DVGSNKGAIIGLMVGGVVIA
Aβ1–42 with novel mutation (P24M)**	DAEFRHDSGYEVHHQKLVFFAED**G**GSNKGAIIGLMVGGVVIA
Aβ1–42 with novel mutation (P22W)***	DAEFRHDSGYEVHHQKLVFFA**M**DVGSNKGAIIGLMVGGVVIA

Note: Mutations or changes are underlined and bold, *medium affinity, **high affinity, ***low affinity.

### Blood Tissue and Plasma Collection Procedures

Ten days after each injection, mice were bled by submandibular phlebotomy using an 18-gauge needle and collected into an EDTA inclusive tube. Plasma was separated by centrifugation 1500 g for 20 minutes with StatSampler from StatSpin (MA). Isolated plasma was frozen at -80°C.

### Antibody Titer Determination

Anti-Aβ antibody (6E10) was purchased from Signet Laboratories (Dedham, MA) and used as a positive control. Antibody levels post-vaccination were assayed via ELISA using Aβ1–42 peptide as the binding antigen. Briefly, 96 well plates were coated with 50 μl Aβ1–42 in cap-binding complex (CBC) buffer (50 mM sodium carbonate, pH 9.6) at 10 μg/ml. A CBC plate is a plate coated with CBC buffer used as a background detection method in order to correct the non-specific binding of sera to the micro plate. A CBC plate was set up as a binding background. Then, both Aβ and CBC coated plates were incubated overnight at 4°C. After 5 washes, plates were subjected to a blocking step with 180 μl blocking buffer (1XPBS containing 1.5% BSA), and incubated for 1 hour at 37°C. Plates were then washed 5 times with wash buffer, and samples diluted with blocking buffer and added to both Aβ and CBC plates at two-fold serial dilutions starting at 1:100. Samples were incubated at 37°C for 1 hour, then subjected to 12 washes with wash buffer. HRP-conjugated anti-mouse IgG (Sigma Aldrich) were loaded into each well at a 1:5000 dilution, incubated for 1 hour at 37°C, then washed 12 times. TMB peroxidase substrate was dissolved in PCB buffer, and 100 μl were added to each well. Colorimetric reactions were stopped with 25 μl 2N H_2_SO_4_. Plates were read at 450 nm/630 nm, and samples with readings 3 times higher than controls were considered positive. The highest dilution was used as the endpoint titer.

### Epitope mapping

Different Aβ peptide fragments (Aβ1–16, 12–28, and 29–42) as well as Aβ1–35 at 20 μg/ml were used to coat a 96-well plate with 50 μl per well. The plate was blocked with 180 μl blocking buffer and blocked for 1 hour at 37°C, then washed 5 times with wash buffer. Pre- and post-immune sera were loaded with serials dilutions. ELISA was conducted using the same protocol described above for the titer assay.

### Cytokine expression detection

The cytokine expression profiles were detected using the Bio-Rad Inc. Bio-Plex kits (Bio-Rad, catalogue # 171F11181). Samples and standards were prepared using company protocols with the initial concentration of standards ranging from 32,000 pg/ml to 1.95 pg/ml. Plasma samples were prepared for analysis by diluting 1 volume of the serum sample with 3 volumes of the Bio-Plex mouse sample diluent. Wells on the 96-well filter plate were pre-wetted with 100 μl of Bio-Plex assay buffer. The buffer was removed by vacuum filtration. The multiplex bead-working solution was vortexed for 15 to 20 sec at medium speed, and 50 μl was pipetted into each well. One-hundred (100) μl of Bio-Plex wash buffer was also pipetted into each well, and then removed by vacuum filtration. Fifty (50) μl of diluted standard was added to wells in the first two columns, and sample was added the remaining wells. The plate was covered with aluminum foil and placed onto a microplate shaker. Samples were incubated for 30 minutes at room temperature.

At the end of the incubation, the reagents were removed by vacuum filtration, and plates were washed 3 times. The Bio-Plex detection antibody working solution was vortexed gently and 25 μl was added to each well. The entire plate was then covered with a new sheet of sealing tape, followed by a sheet of foil. The plate was then incubated at room temperature with shaking for 30 minutes. Afterward, the sealing tape was removed and the liquid extracted by vacuum filtration. This was followed by 3 washes, with blotting in between each wash.

One-x (1x) streptavidin-PE was vigorously vortexed, and 50 μl pipetted into each well. The plate was again covered with sealing tape and foil, then incubated at room temperature with shaking for 10 minutes. After incubation, the sealing tape was again removed, the liquid extracted by vacuum filtration, and 3 wash steps with blotting in between were performed. The beads were then re-suspended in each well with 125 μl of Bio-Plex assay buffer. The plate was again covered with a new sheet of sealing tape and incubated at room temperature with shaking for 30 seconds.

Finally, the plates were read. Because of the naturally-occurring variability of cytokine levels, optical density readings for each cytokine were normalized to a 0–1 scale that was used to compare animal groups.

### IgG isotyping

To further confirm the inflammation and the contribution of cytokines to Ig subclass switching modulation, we detected Ig isotyping by using the Beadlyte^® ^Mouse Immunoglobulin Isotyping Kit by Upstate Cell Signaling Solutions (Temecula, CA), and following manufacturer's instructions. Total Ig isotyping was assayed instead of anti-Aβ-specific antibody because any Ig difference in the same mouse is due to the antigen stimulation. In addition, this method allows the monitoring of overall Ig change pre- and post-vaccination. This method produces an IgG1/IgG2a ratio and this ratio helps to differentiate Th1 or Th2 responses in vaccinated mice. Because IgG1 is driven by IL-4 (Th2), and IgG2a is driven by IFN-γ (Th1) [35], an increase in post-vaccination ratio indicates a Th2 response, and a decrease in post-vaccination ratio indicates a Th1 response.

### Immunostaining

To evaluate antibodies generated from BALB/c mice, cross-reaction to human Aβ was evaluated in tg mouse brain tissue. Tg mice were euthanized with an overdose of anesthesia, brain blood was removed by intracardial perfusion, and brain tissue was harvested as per established protocol [46]. Immunostaining assay was completed as previously described by Nilsson et al. [46].

### Statistical analysis

All data were analyzed using analysis of variance (ANOVA). After interpreting the results of the ANOVA models, post-hoc differences between groups (pair-by-pair differences) were assessed using Fisher's Least Significant Differences (LSD) test.

## Authors' contributions

CC conceived the design of the study, conducted data analysis, and served as primary writer of the manuscript. XL prepared the vaccines and injected the mice, and performed all sample analysis and collection. MMW assisted in restructuring the manuscript, improving writing quality, providing additional references for citation, and including a public health perspective. EAJ was responsible for assisting in manuscript preparation, compilation, editing, and formatting for journal submission. HP guided data interpretation and proofreading, and contributed resources from his laboratory. All authors read and approved the final manuscript.
